# Application of Surgical Apgar Score in intracranial meningioma surgery

**DOI:** 10.1371/journal.pone.0174328

**Published:** 2017-04-06

**Authors:** Shih-Yuan Hsu, Chien-Yu Ou, Yu-Ni Ho, Yu-Hua Huang

**Affiliations:** 1 Department of Neurosurgery, Kaohsiung Chang Gung Memorial Hospital and Chang Gung University College of Medicine, Kaohsiung, Taiwan; 2 Department of Surgery, Kaohsiung Armed Forces General Hospital, Kaohsiung, Taiwan; 3 Department of Emergency Medicine, Kaohsiung Chang Gung Memorial Hospital and Chang Gung University College of Medicine, Kaohsiung, Taiwan; George Washington University, UNITED STATES

## Abstract

Surgical resection is the main therapeutic option for intracranial meningiomas, but it is not without significant morbidities. The Surgical Apgar Score (SAS), assessed by intraoperative blood pressure, heart rate, and blood loss, was developed for prognostic prediction in general and vascular surgery. We aimed to examine whether the application of SAS in patients undergoing craniotomy for meningioma resection can predict postoperative major complications. We retrospectively enrolled 99 patients that had undergone intracranial meningioma surgery. The patients were subdivided into 2 groups based on whether major complications were present (N = 34) or not (N = 65). We recognized the intergroup differences in SAS and clinical variables. The incidence of 30-day major complications in patients after operation was 34.3%. The lengths of ICU and hospital stay for the morbid cases were prolonged significantly (p = 0.009, p < 0.001, respectively). In the multivariate logistic regression model, SAS was an independent predicting factor of major complications following surgery for intracranial meningiomas (odds ratio, 95% confidence interval = 0.57, 0.38–0.87; p = 0.009), and thus a decrease of one mean SAS increased the rate of major complications by 43%. In conclusions, SAS is an independent predictor of major complications in patients undergoing intracranial meningioma surgery, and provides acceptable risk discrimination. Since this scoring system is relatively simple, objective, and practical, we suggest that SAS be included as an indicator in the guidance for the level of care after craniotomy for meningioma resection.

## Introduction

Meningiomas are composed of neoplastic arachnoidal cells imbedded in the meninges, and constitute 13%–26% of primary intracranial tumors [1, 2]. Most meningiomas are slowly growing and benign, and tend to compress the adjacent structures rather than infiltrate them. Because of the relatively clear operative plane, surgery aimed at total resection of the tumors is the main therapeutic option. While surgical removal of intracranial meningiomas can be curative and allow timely reduction of the mass effect, it is not without significant adverse events. Particularly, the incidence of meningiomas peaks after the fifth decade of life, and elderly patients are more likely to have complications following surgery [3, 4]. Thus the ability to identify the immediate postoperative state and determine a patient’s risk of complications is quite important, and can guide the level of care and alleviate the effect of morbidities.

In 2007, Gawande et al. introduced the Surgical Apgar Score (SAS) to predict the occurrence of major postoperative morbidities and mortality after general and vascular surgery [5]. SAS is a 10-point score based on 3 easily obtained parameters: the estimated blood loss, lowest heart rate, and lowest mean arterial pressure during surgery (Table 1). This scoring system has been validated more broadly for use in several cohorts of patients undergoing orthopedic, gynecologic, traumatic, urologic, or colorectal surgery [6, 7, 8]. In addition, Ziewacz et al. showed that the use of SAS in a general neurosurgical population can allow risk stratification [9]. However, the diversity of the neurosurgical field, from emergency to elective or brain to spinal surgery, should be taken into consideration, and the efficacy of SAS in the setting of each procedure should be accessed.

**Table 1:** The 10-Point Surgical Apgar Score

	*No*. *of Points*^a^				
	*0*	*1*	*2*	*3*	*4*
*Estimated blood loss*, *ml*	*>1000*	*601–1000*	*101–600*	≦*100*	─
*Lowest mean arterial pressure*, *mmHg*	*<40*	*40–54*	*55–69*	≧*70*	─
*Lowest heart rate*, *beats per min*	*>85*	*76–85*	*66–75*	*56–65*	*≦55*^b^

a The Surgical Apgar Score is calculated at the end of operation and is the sum of the points from each category.

b Occurrence of pathologic bradyarrhythmia, including sinus arrest, atrioventricular block or dissociation, junctional or ventricular escape rhythms, and asystole, also receives 0 points for lowest heart rate.

Whether SAS applied with patients undergoing intracranial meningioma surgery differentiates and predicts prognosis remains to be elucidated. In the present study, we collected clinical data and quantified the relationship between SAS and major complications after craniotomy for meningioma resection.

## Materials and methods

### Data collection

This study was retrospectively conducted at Kaohsiung Chang Gung Memorial Hospital, a 2686-bed tertiary referral institute in Taiwan. After being approved by the institutional review board, we reviewed the documents of patients that had undergone craniotomy for intracranial meningioma resection from February 2009 through December 2013. Patients who were treated for recurrent tumors or tissue biopsy alone were excluded. A total of 99 cases were included for assessment. The research staff collected clinical information, consisting of the demographic data, presenting symptoms/signs, preoperative laboratory examinations, Karnofsky Performance Scale (KPS) score, and American Society of Anesthesiologists (ASA) Physical Status Classification. Details of the operations for the calculation of SAS, including intraoperative blood loss, lowest heart rate, and lowest mean arterial pressure, were recorded from computerized or paper medical documents.

### Image evaluation

Magnetic resonance imaging (MRI) features of the brain were acquired prior to surgery; they basically included T1, T2 and T1 sequences with gadolinium enhancement. The locations of the tumors were categorized as convexity, parasagittal/falx, cranial base, or posterior fossa. A critical location was defined as a location in which a tumor was attached to primary vascular or nervous structures, such as an eloquent area or the cranial base. We calculated the size of the tumor by measuring the largest diameter of the lesion. The presence of peritumoral edema was identified on T2-weighted images, and classified as absent, moderate (peritumoral only), or severe (with a shift of midline structures). For routine postoperative evaluation or in the event of a new onset of neurological deficits, follow-up computed tomography (CT) or MRI scans were performed.

### Clinical management

Some of the patients received preoperative embolization for meningiomas per the surgeon's decision based on the size, location, and blood supply of the tumors. All the patients underwent craniotomy for removal of meningiomas, and intraoperative navigator guidance, microscopic assistance, or electrophysiological monitoring was selectively used as adjuncts to surgical resection. All specimens were obtained to establish a histological diagnosis, and the tumors were subdivided according to the World Health Organization’s classification [10]. The extent of surgery was accessed using the Simpson grade of resection score [11]. The patients received postoperative monitoring and treatment in the intensive care unit (ICU), and had intubation with ventilator assistance for different durations, depending on the neurological and medical recovery.

### Outcome assessment

The main outcome for this study was 30-day major complications after intracranial meningioma surgery. The following events were defined as major complications [5]: myocardial infarction, cardiac arrest requiring cardiopulmonary resuscitation, ventilator use for 48 hours or longer, unplanned intubation, pulmonary embolism, pneumonia, coma for 24 hours or longer, stroke, sepsis, septic shock, deep or organ-space surgical site infection, wound disruption, systemic inflammatory response syndrome, deep venous thrombosis, acute renal failure, and bleeding requiring > 4 U red cell transfusion within 72 hours after operation. All-cause mortality was considered a major complication, but urinary tract infection or superficial surgical site infection was not.

### Statistical analysis

Data were analyzed by SPSS (IBM SPSS Statistics, version 20.0). Descriptive statistics were showed as frequencies (%) or as mean and standard deviation (SD). Continuous parameters were assessed using the Student’s *t*-test or Mann–Whitney *U*-test. Categorical parameters were compared using the chi-square test or Fisher’s exact test. All parameters with a p value < 0.05 were entered into multivariable logistic regression to adjust for independent predicting factors of postoperative major complications. The results were demonstrated as odds ratios and 95% confidence intervals. A p value of < 0.05 was considered to be significant statistically.

## Results

### Baseline characteristics

The 99 patients who underwent craniotomy for intracranial meningiomas included 40 males and 59 females. The mean age was 60.9 (SD, 13.8; range, 20–87) years. Underlying medical conditions included 25 cases of diabetes mellitus, 44 of hypertension, 3 of coronary artery disease, 10 of previous stroke, and 6 patients undergoing antiplatelet therapy. At admission, the average KPS score was 68.0 (SD, 13.8; range, 20–90). The number of patients with ASA physical status classification I, II, III, and V was 3, 37, 58, and 1, respectively. The MRI scans showed 38 convexity, 22 parasagittal/falx, 27 cranial base, and 12 posterior fossa meningiomas. There were 62 tumors in a critical location. The degree of peritumoral edema was as follows: absent in 40 patients, moderate in 40 patients, and severe in 19 patients. The mean size of the tumor was 4.6 (SD 1.8; range, 1–9) cm in maximal diameter. Thirty-one patients had preoperative transarterial embolization of meningiomas to facilitate surgery. The number of patients with Simpson resection grade 1, 2, 3, 4, and 5 were 14, 56, 12, 15, and 2, respectively. The mean duration of operation was 9.6 (SD, 3.4; range 3.9–21.1) hours. Sixty-eight patients received intraoperative red cell transfusion. The length of postoperative ICU stay and overall hospital stay on average were 5.6 (SD 6.4; range 2–59) and 19.6 (SD 11.2; range 7–68) days, accordingly.

### Analysis of surgical apgar scores

The mean blood loss during operation was 807.0 (SD, 806.3; range 20–4200) ml. The lowest mean arterial pressure on average was 59.6 (SD, 9.1; range 36–86) mmHg. The lowest heart rate ranged from 38–96 beats/min, and the mean value was 59.8 (SD, 9.7) beats/min. Assessment of the 3 intraoperative parameters showed 6 patients (6.1%) with a SAS of 0–3, 51 (51.5%) with a SAS of 4–6, and 42 (42.4%) with a SAS of 7–10. The mean SAS was 6.0 (SD, 1.6; range 1–9).

### Thirty-day major complications after operation

Major complications were documented in 34 of the 99 patients within 30 days after intracranial meningioma surgery, and the overall incidence was 34.3%. The causes and cause-specific frequency of the complications included 11 (11.1%) with ventilator use for 48 hours or longer, 7 (7.1%) with pneumonia, 7 (7.1%) with bleeding requiring > 4 U red cell transfusion within 72 hours after operation, and others (Table 2). There was no 30-day or in-hospital mortality. The mean lengths of postoperative ICU stay and hospital stay for patients with complications were both longer than those for patients without complications (p = 0.009, p < 0.001, respectively). The mean KPS score at discharge was significantly lower among the morbid patients (p < 0.001) (Table 3).

**Table 2:** The causes and cause-specific frequency of major complications

	*All cases*
	*N = 99*
*Complications*	*N (%)*
*Red cell transfusion > 4U within 72 hr after operation*	*7 (7*.*1)*
*Deep venous thrombosis*	*1 (1*.*0)*
*Ventilator use > 48 hr*	*11 (11*.*1)*
*Pneumonia*	*7 (7*.*1)*
*Stroke*	*4 (4*.*0)*
*Wound disruption*	*3 (3*.*0)*
*Deep or organ-space surgical site infection*	*4 (4*.*0)*
*Sepsis*	*6 (6*.*1)*
*Systemic inflammatory response syndrome*	*3 (3*.*0)*
*Death*	*0 (0)*

**Table 3:** Comparisons of clinical characteristics in patients with or without major complications after intracranial meningioma surgery

	*Total cases*	*With complication*	*Without complication*	
	*N = 99*	*N = 34*	*N = 65*	*P value*
*Age (year)*	*60*.*9 (SD 13*.*8)*	*62*.*5 (SD 15*.*9)*	*60*.*0 (SD 12*.*6)*	*0*.*39*
*Gender (male)*	*40 (40*.*4%)*	*18 (52*.*9%)*	*22 (33*.*8%)*	*0*.*07*
*Underlying medical condition*				
* Diabetes mellitus*	*25 (25*.*3%)*	*10 (29*.*4%)*	*15 (22*.*1%)*	*0*.*49*
* Hypertension*	*44 (44*.*4%)*	*17 (50*.*0%)*	*17 (26*.*2%)*	*0*.*42*
* Coronary artery disease*	*3 (3*.*0%)*	*1 (2*.*9%)*	*2 (3*.*1%)*	*1*.*00*
* Stroke*	*10 (10*.*1%)*	*3 (8*.*8%)*	*7 (10*.*8%)*	*1*.*00*
* Antiplatelet therapy*	*6 (6*.*1%)*	*3 (8*.*8%)*	*3 (4*.*6%)*	*0*.*41*
*Symptom/sign*				
* Headache*	*38 (38*.*4%)*	*12 (35*.*3%)*	*26 (40*.*0%)*	*0*.*65*
* Vomiting*	*15 (15*.*2%)*	*5 (14*.*7%)*	*10 (15*.*4%)*	*0*.*93*
* Blurred vision*	*8 (8*.*1%)*	*2 (5*.*9%)*	*6 (9*.*2%)*	*0*.*71*
* Extremity weakness*	*28 (28*.*3%)*	*11 (32*.*4%)*	*17 (26*.*2%)*	*0*.*52*
* Aphasia*	*7 (7*.*1%)*	*3 (8*.*8%)*	*4 (6*.*2%)*	*0*.*69*
* Seizure*	*15 (15*.*2%)*	*4 (11*.*8%)*	*11 (16*.*9%)*	*0*.*50*
* Mental change*	*14 (14*.*1%)*	*8 (23*.*5%)*	*6 (9*.*2%)*	*0*.*07*
*Duration of symptom/sign (month)*	*5*.*3 (SD 6*.*4)*	*5*.*4 (SD 6*.*4)*	*5*.*3 (SD 6*.*5)*	*0*.*97*
*Preoperative laboratory data*				
* Hemoglobin (g/dl)*	*13*.*0 (SD 1*.*8)*	*12*.*7 (SD 2*.*0)*	*13*.*2 (SD 1*.*7)*	*0*.*27*
* White blood cell (count/μL)*	*7897*.*0 (SD 3431*.*6)*	*7644*.*1 (SD 3919*.*7)*	*8029*.*2 (SD 3171*.*5)*	*0*.*60*
* Platelet (count/μL)*	*227*.*3 (SD 66*.*6)*	*208*.*3 (SD 63*.*0)*	*237*.*2 (SD 66*.*7)*	*0*.*04*
* Prothrombin time INR*^a^	*1*.*0 (SD 0*.*1)*	*1*.*0 (SD 0*.*1)*	*1*.*0 (SD 0)*	*0*.*08*
*KPS score*^b^ *at admission*	*68*.*0 (SD 13*.*8)*	*62*.*9 (SD 18*.*3)*	*70*.*6 (SD 9*.*8)*	*0*.*04*
*ASA classification*^c^ *> II*	*59 (59*.*6%)*	*23 (67*.*6%)*	*36 (55*.*4%)*	*0*.*24*
*Location of tumors*				*0*.*02*
* Convexity*	*38 (38*.*4%)*	*8 (23*.*5%)*	*30 (46*.*2%)*	
* Parasagittal / Falx*	*22 (22*.*2%)*	*13 (38*.*2%)*	*9 (13*.*8%)*	
* Cranial base*	*27 (27*.*3%)*	*8 (23*.*5%)*	*19 (29*.*2%)*	
* Posterior fossa*	*12 (12*.*1%)*	*5 (14*.*7%)*	*7 (10*.*8%)*	
*Critical location*	*62 (62*.*6%)*	*25 (73*.*5%)*	*37 (56*.*9%)*	*0*.*11*
*Preritumoral edema*				*0*.*36*
* Absent*	*40 (40*.*4%)*	*12 (35*.*3%)*	*28 (43*.*1%)*	
* Moderate*	*40 (40*.*4%)*	*17 (50*.*0%)*	*23 (35*.*4%)*	
* Severe*	*19 (19*.*2%)*	*5 (14*.*7%)*	*14 (21*.*5%)*	
*Size of tumors (cm)*	*4*.*6 (SD 1*.*8)*	*5*.*2 (SD 1*.*8)*	*4*.*3 (SD 1*.*8)*	*0*.*03*
*WHO classification*^d^ *of tumors > I*	*29 (29*.*3%)*	*9 (26*.*5%)*	*20 (30*.*8%)*	*0*.*66*
*Preoperative tumor embolization*	*31 (31*.*3%)*	*12 (35*.*3%)*	*19 (29*.*2%)*	*0*.*54*
*Intraoperative red cell transfusion*	*68 (68*.*7%)*	*29 (85*.*3%)*	*39 (60*.*0%)*	*0*.*01*
*Duration of operation (hour)*	*9*.*6 (SD 3*.*4)*	*10*.*2 (SD 3*.*9)*	*9*.*4 (SD 3*.*0)*	*0*.*28*
*Simpson grade of tumor resection*	*2*.*3 (SD 1*.*0)*	*2*.*7 (SD 1*.*0)*	*2*.*2 (SD 0*.*9)*	*0*.*01*
*Surgical Apgar Score*	*6*.*0 (SD 1*.*6)*	*5*.*1 (SD 1*.*6)*	*6*.*5 (SD 1*.*3)*	*<0*.*001*
*Length of ICU*^e^ *stay (day)*	*5*.*6 (SD 6*.*4)*	*8*.*8 (SD 10*.*2)*	*3*.*9 (SD 1*.*2)*	*0*.*009*
*Length of hospital stay (day)*	*19*.*6 (SD 11*.*2)*	*28*.*0 (SD 14*.*7)*	*15*.*2 (SD 5*.*0)*	*<0*.*001*
*KPS score at discharge*	*72*.*8 (SD 14*.*5)*	*62*.*4 (SD 16*.*9)*	*78*.*3 (SD 9*.*3)*	*<0*.*001*

a. International normalized ratio

b. Karnofsky Performance Scale Score

c. American Society of Anesthesiologists Physical Status Classification

d. World Health Organization’s Classification

e. Intensive care unit

### Predictors for major complications

In a comparison of clinical features of patients with or without major complications, statistical analysis identified the following 7 parameters with a p value < 0.05: preoperative platelet count (p = 0.04), KPS score at admission (p = 0.04), location of tumors (p = 0.02), size of tumors (p = 0.03), intraoperative red cell transfusion (p = 0.01), Simpson grade of tumor resection (p = 0.01), and SAS (p < 0.001) (Table 3). All of these factors were entered into multivariable regression analysis, and SAS was the only independent predictor for major complications after surgery for intracranial meningiomas (odds ratio, 95% confidence interval = 0.57, 0.38–0.87; p = 0.009) (Table 4). Thus a decrease of one mean SAS increased the rate of major complications by 43%.

**Table 4:** Multivariable analysis for independent predictors of major complications after intracranial meningioma surgery

	*Major complication*	
	*Odds ratio (95% CI)*	*P value*
*Preoperative platelet count*	*0*.*99 (0*.*99–1*.*00)*	*0*.*21*
KPS score* *at admission*	*0*.*96 (0*.*93–1*.*00)*	*0*.*07*
*Location of tumors*		
* Convexity*	*Reference*	*0*.*33*
* Parasagittal / Falx*	*2*.*62 (0*.*64–10*.*74)*	*0*.*18*
* Cranial base*	*0*.*86 (0*.*20–3*.*74)*	*0*.*84*
* Posterior fossa*	*2*.*41 (0*.*42–13*.*67)*	*0*.*32*
*Size of tumors*	*1*.*27 (0*.*91–1*.*77)*	*0*.*16*
*Intraoperative red cell transfusion*	*0*.*96 (0*.*22–4*.*20)*	*0*.*96*
*Simpson grade of tumor resection*	*1*.*32 (0*.*72–2*.*42)*	*0*.*37*
*Surgical Apgar Score*	*0*.*57 (0*.*38–0*.*87)*	*0*.*009*

* Karnofsky Performance Scale Score

### Relationship between SAS and major complications

**Fig 1:**
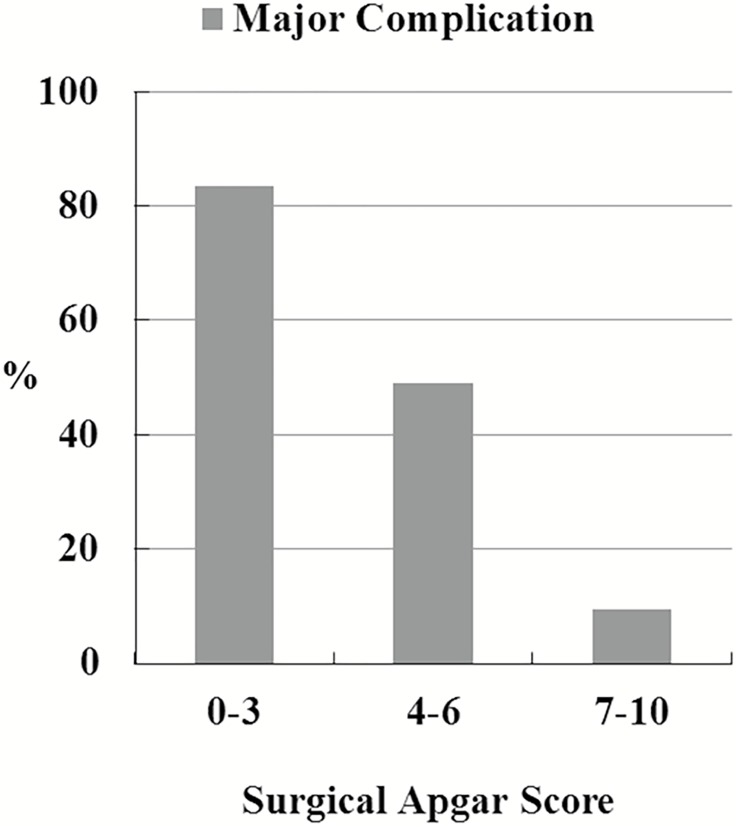
Surgical Apgar Score versus major complications.

**Fig 2:**
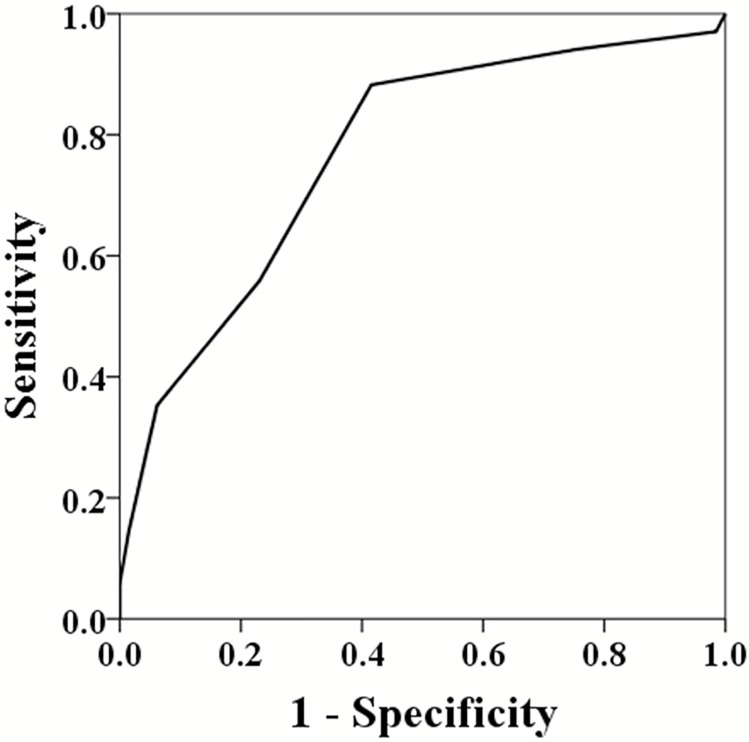
Receiver operating characteristic curve for Surgical Apgar Score as a predictor of major complications (area under the curve = 0.768).

We stratified the patients into 3 subgroups based on SAS (0–3, 4–6, and 7–10). Major complications occurred in 5 of the 6 patients (83.3%) with SAS 0–3, 25 of the 51 (49.0%) with SAS 4–6, and 4 of the 42 (9.5%) with SAS 7–10 (Fig 1). Using the receiver operating characteristic (ROC) curve for SAS as a predictor of major complications after intracranial meningioma surgery, the area under the ROC curve was 0.768, which represented acceptable discriminatory power of SAS (Fig 2).

## Discussion

The extended life expectancy and increased use of neuroimaging facilities in current clinical practice have contributed to the diagnosis and treatment of an increasing proportion of intracranial meningiomas, particularly in the elderly [2, 3, 4]. Since more and more attention is being given to patient safety, the complications of intracranial meningioma surgery should be under closer investigation. In the review and meta-analysis conducted by Poon et al. [4], the complication rates of elderly patients following meningioma resection ranged from 2.7% to 29.8%, and the overall incidence of complications was 20% per patient (range, 3–61%). In this study, the rate of 30-day major complications after surgical removal of tumors was more than 30%. The diversity of the definition of complications may explain why the incidence is highly variable in published studies. In addition, the mean size of the tumors was 4.6 cm and about 40% of the tumors located in cranial base and posterior fossa of our cases. Because larger and complex meningiomas are potentially deeply-seated or encase nerves and vessels, their safe resection theoretically takes more time. These variables probably contribute to higher transfusion rate and more morbidities. As expected, these morbid patents have a concomitantly prolonged length of ICU or hospital stay, and also suffer from the worse surgical results.

An increased understanding of the risks of postoperative morbidities is pivotal to improving therapeutic outcomes. In addition, careful patient stratification offers an opportunity to control medical costs. Several risk factors for complications or mortality among patients with intracranial meningioma surgery, such as age, sex, functional status, ASA physical status classification, preoperative disseminated cancer, tumor location, peritumoral edema, tumor size, or extent of tumor removal, have been documented [3, 4, 12, 13]. Furthermore, we expanded and tested SAS as a means for predicting major complications after meningioma resection. The results showed SAS was a meaningful variable in multivariable logistic regression. The differences in the observed rates of complications between patients, from the lowest (0–3) and the highest (7–10) score groups, were up to 73.8%, which showed the discriminative ability of SAS.

SAS was derived from a retrospective analysis of 303 patients undergoing colectomy, and validated in 2 prospective cohorts: 102 colectomy patients and 767 patients undergoing general or vascular operations [5]. Like the concept of the Apgar score for newborns, SAS provides immediate feedback and communication regarding the patient's condition for clinicians. Several models, including the Acute Physiology and Chronic Health Evaluation (APACHE) score, Physiologic and Operative Severity Score (POSSUM), and Therapeutic Intervention Scoring System-28 (TISS-28), have also been adapted or developed to predict general postoperative outcomes [14, 15, 16]. Even with their high predictive value, these scores are not widely used for surgical patients because of the need for numerous parameters, dependence on laboratory examinations, or difficulty in rapid calculation. SAS greatly benefits from the sum of 3 easily available intraoperative data points, and has been proved to predict surgical outcome independent of preoperative physiological status [5, 6]. Some grading systems, such as CRGS (Clinical–Radiological Grading System), the SKALE score (sex, Karnofsky, ASA classification, location, edema), or GSS (geriatric scoring system) were specifically constructed to predict prognosis after intracranial meningioma resection [13, 17, 18]. Nevertheless, they primarily aim to standardize surgical indications, but are unable to offer advanced and timely information for postoperative care. In addition, these grading systems emphasize elderly patients harboring meningiomas, and have difficulty in reproducing their predictive ability when extended to younger patients [19]. In comparison, SAS is not confined by cut-off values of parameters, and can be applied in a population with a wide age distribution.

This study is one of a few investigations of SAS that focus on the neurosurgical field. Urrutia et al. reported SAS allowed risk stratification and had good discriminatory power in 268 patients undergoing spinal surgery [20]. In a retrospective study of a general neurosurgical cohort, Ziewacz et al. concluded the application of SAS predicted 30-day postoperative mortality and complication rates as well as extended intensive care unit and hospital stay [9]. Their results were convincing, but the study population included emergency, traumatic, spinal, and intracranial cases. In our opinion, SAS may discriminate the risks between elective and emergency, non-traumatic and traumatic, or spinal and craniotomy surgeries. Whether SAS can be used with each condition and procedure, such as craniotomy for resection of intracranial meningiomas, should be clarified. In this series, we further confirmed that this scoring system was useful and practical as a predictive tool for the examination of patients undergoing meningioma resection.

Reynolds et al. broadened the use of SAS to many surgical subspecialties after a review of more than 120,000 patients, but the strength of the correlation varied [7]. Moreover, SAS appeared to have a limited role in the management of individual patients after orthopedic surgery and elective general/vascular surgery [21]. We considered that the variability of blood loss in individual surgical subspecialties or procedures may be one of the determinants for the utility of SAS. Craniotomy for meningioma removal is frequently accompanied with substantial blood loss, and may require perioperative blood transfusion. In this series, the mean blood loss in intracranial meningioma surgery was 807 ml, with a range that varied from 20–4200 ml. Acute blood loss also resulted in potential changes in heart rate and arterial blood pressure. As a result, this scoring system was able to subdivide our patients into groups with different risks of complications.

SAS is an objective and simple prognosticator for surgery. Lower scores are accompanied with higher rates of major complications, and this relationship allows for assessment of quality improvement plans. If a reduction in the frequency of patients with lower scores produces a reduction in the incidence of morbidities, high score levels may be a modifiable aim for patient safety in surgical resection of intracranial meningiomas. The future investigation on the ability of SAS to modify rather than predict postoperative outcomes is needed to verify its advanced use.

Our study design has some potential limitations. A retrospective analysis of preexisting data suffers from the inherent restrictions of such researches. Data collection is less precise and less complete than planned study. Surgical blood loss was an estimate calculated as the sum of blood in suction containers and soaked gauzes, and the true lowest heart rate or arterial pressure may be not accurately captured from computerized or paper charts. The number of patients was still relatively small from a statistical standpoint, and this investigation may be underpowered to discover some significant risk factors. Additionally, our results reflect the experience of an individual medical institute; thus, the findings may not be representative of all cases undergoing meningioma resection in other hospitals. Even with the issue in the preliminary analysis, we consider that these data provide valuable information for postoperative care and possible routes for disease or procedural modification.

## Conclusions

SAS is an independent predictor of major complications in patients undergoing intracranial meningioma surgery, and provides acceptable risk discrimination. Because the scoring system is relatively simple, objective, and practical, we suggest that SAS be included as an indicator in the guidance for the level of care after craniotomy for meningioma resection.

## Supporting information

S1 File The raw data used in the paper. (XLSX)
